# Supplementary material for: Sex-specific behavioral feedback modulates sensorimotor processing and drives flexible social behavior
Source: Nat Commun. 2026 May 4;17:4026. doi: 10.1038/s41467-026-72057-9 (PMC13139495; doi:10.1038/s41467-026-72057-9)
Supplement: Supplementary file 1 — Supplementary Information [file 41467_2026_72057_MOESM1_ESM.pdf]

Supplementary Figures

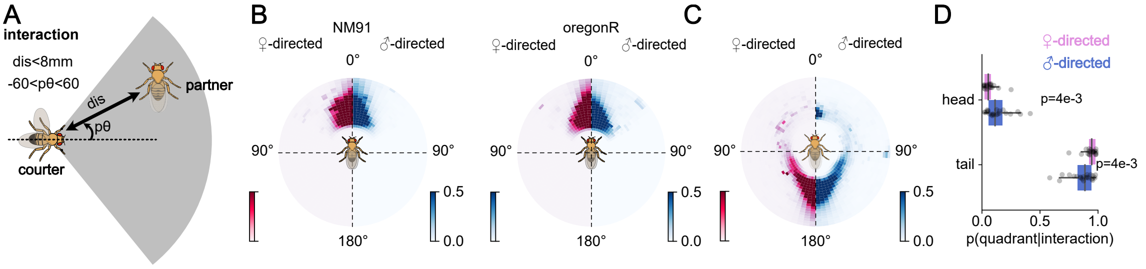

Figure S1: Fly orientation during interactions.
A Definition of interaction, courter (or focal) and partner (or target) flies. An interaction occurs when the flies are within 8 mm of each other and one fly (the partner or target) is within the field of view (±60°) of the other fly (courter). When both flies are within a field of view of less than ±60° of each other, the fly that was initially courter remained so.
B Position of the female and male partner fly with respect to an NM91 (left) or OregonR (right) courter male. The courter fly is always oriented towards the partner during interactions.
C Position of an OregonR courter male around a female (left, magenta) or a male (right, blue) OregonR partner during interactions. 0° and 180° represent the partner's head and tail, respectively.
D Fraction of time spent by the OregonR courter male near the head and tail of the OregonR partner fly during interactions. Dots represent individual fly pairs (N=20 male-female and 22 male-male pairs). Central mark indicates the median; the bottom and top edges of the box indicate the 25th and 75th percentiles, respectively. Whiskers extend to 1.5 times the interquartile range away from the box edges. p-values were computed using a two-sided Mann-Whitney U test.

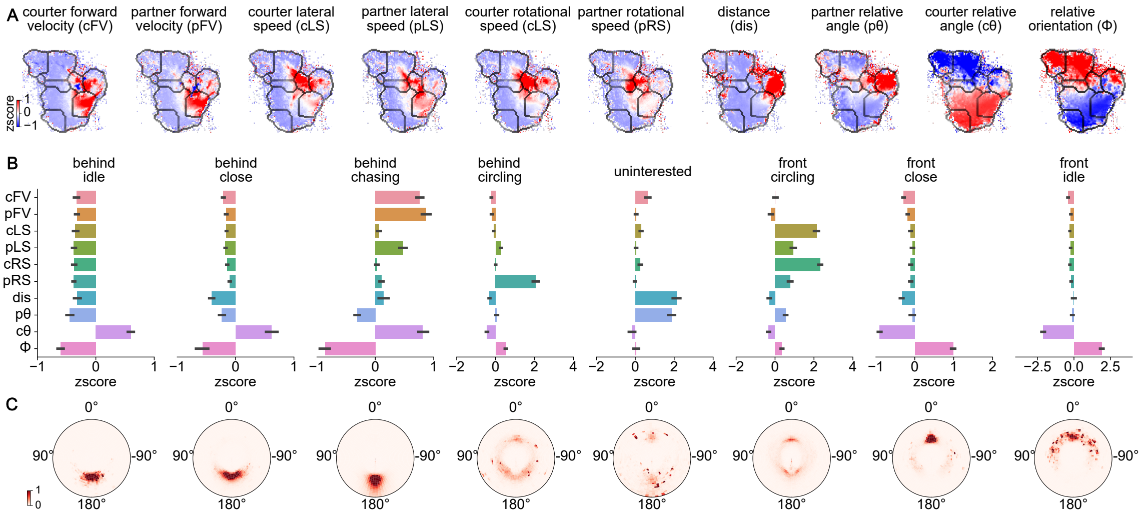

Figure S2: Unsupervised quantification of male- and female-directed interactions.
A Behavioral features for different locations in the social map. Color shows the z-scored behavioral feature values for each pixel in the map.
B Behavioral features in each of the eight social modes. Bars and error bars show mean ±standard deviation across fly pairs. Features were z-scored.
C Position of the courter around the partner during each of the eight social modes. In the first four modes (behind idle, behind close, behind chasing, behind circling), the courter is mostly placed behind the partner. For the last three modes (front circling, front close, and front idle), the courter is mostly placed near the partner's head. The radial axis is normalized to the target length and is limited to three fly lengths.

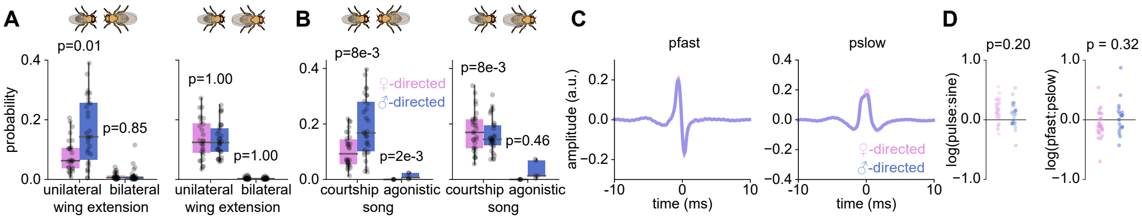

Figure S3: Female and male-directed singing were courtship-like with little difference in short-term song characteristics.

A Unilateral and bilateral wing extensions during tail (left) and head interactions (right) with a female (magenta) and male (blue) partner.

B The amount of courtship and agonistic song during tail (left) and head interactions (right).

C The average waveform of fast (Pfast: N=23288 for female-directed and 24913 for male-directed) and slow pulses (Pslow: N=21329 for female-directed and 21374 for male-directed) sung towards female and male partners. The pulse shape does not change with partner sex.

D Pulse-to-sine (left) and fast-to-slow pulse (right) ratio during female- and male-directed interactions.

p-values are computed using a two-sided Mann-Whitney U test. Dots in A, B, and D correspond to the average value for each fly pair (N=5 pairs each of male-female and male-male agonistic song. For everything else, N=30 pairs each of male-female and male-male). For A, B, central mark indicates the median; the bottom and top edges of the box indicate the 25th and 75th percentiles, respectively. Whiskers extend to 1.5 times the interquartile range away from the box edges.

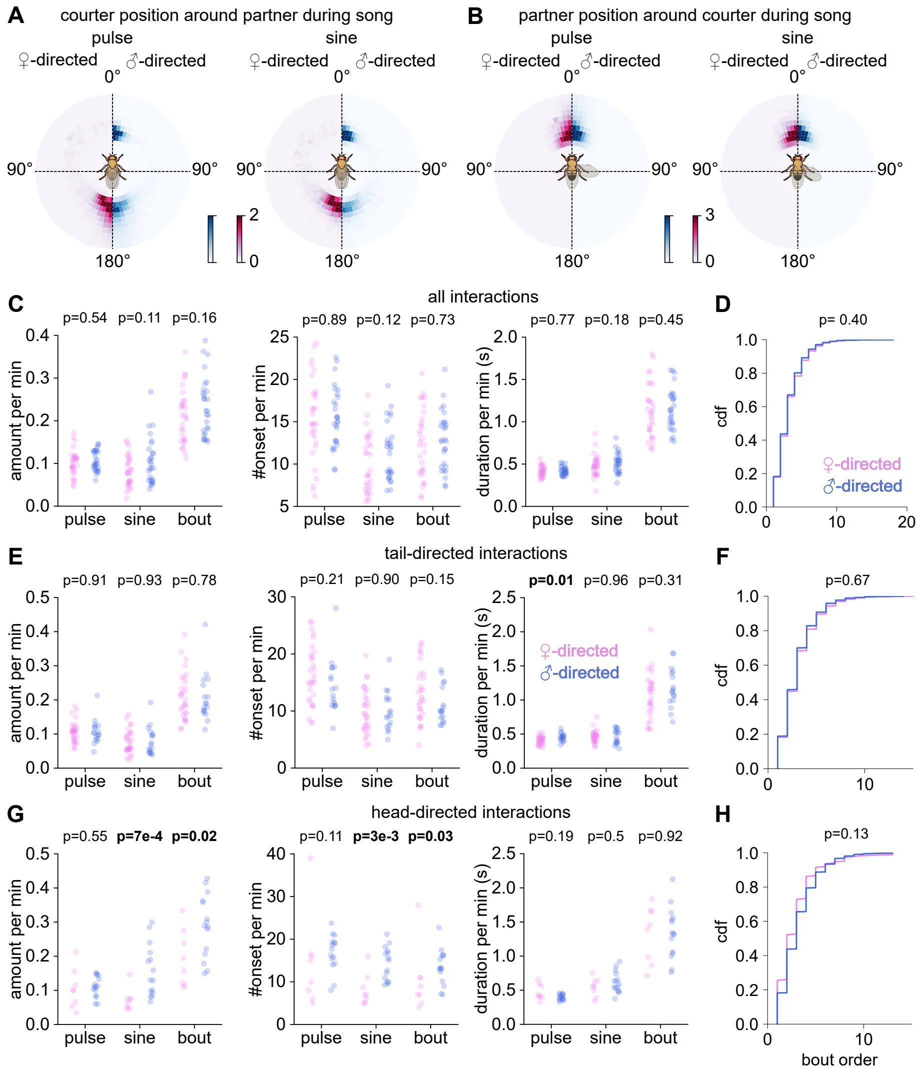

Figure S4: Song patterns differ most strongly during head interactions.
A Courter position around the partner during singing pulse (left) and sine (right) song. The courter is situated mostly near the tail when singing to a female, but also near the head when singing to a male. The courter is situated at a broader range of distances and angular positions when interacting near the tail of a female than the tail of a male.
B Partner position around courter during pulse (left) and sine (right) song. Both songs are produced when the partner is within a narrow field of view of the courter (narrower during male-directed singing). **C** Amount, number of onsets, and duration of pulse, sine, song bouts per minute during male- (blue) and female-directed (magenta) interactions. The overall difference between female- and male-directed song patterning statistics is small. Dots correspond to the average value of song metrics for each fly pair (N=30 pairs each of male-female and male-male).
D Cumulative density functions of the bout order for male (blue) and female-directed (magenta) song. Bout order corresponds to the number of transitions between song modes (pulse, sine) within a song bout.
E–F Same as C and D restricted to tail interactions. Dots correspond to the average value of song metrics for each fly pair with tail-directed interactions (N=30 pairs each of male-female and male-male).
G–H Same as C and D restricted to head interactions. Dots correspond to the average value of song metrics for each fly pair with head-directed interactions (N=8 pairs of male-female and 15 pairs of male-male)
All p-values are computed using a two-sided Mann-Whitney U test.

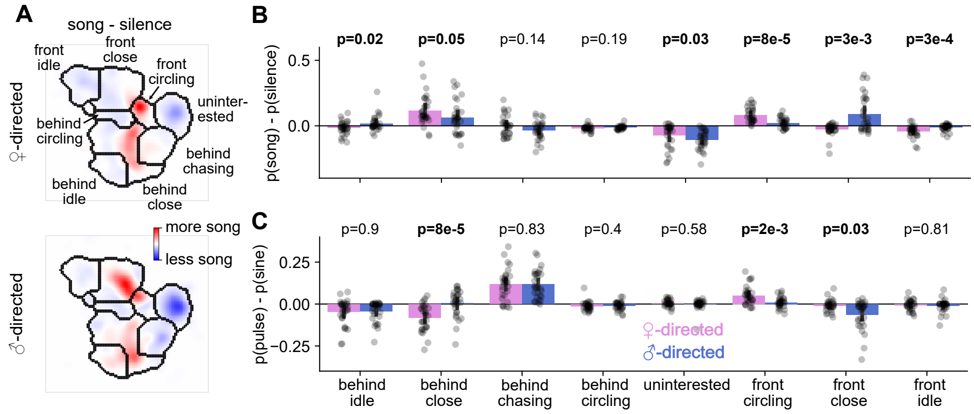

Figure S5: Males sing in different social modes towards two partners
A Difference between the interaction state spaces conditioned to song (pulse or sine) and silence during female- (top) and male-directed (bottom) interactions showing context-specific differences in singing based on partner sex. When interacting with females, males produce more song during the close, chasing, and frontal circling modes. When interacting with males, males sing more during the behind and idle, behind and close, and frontal close modes.
B, C Difference between the probabilities of singing and staying silent (B, data from A) and pulse and sine (L, data from Fig. 2F–G) in different social modes. Dots correspond to average values for each fly pair (N=30 pairs each of male-female and male-male). Bar heights and error bars correspond to the average ±std across fly pairs.
All p-values are computed using a two-sided Mann-Whitney-U test. For B and C, bar heights represent mean and errorbars represent s.d.

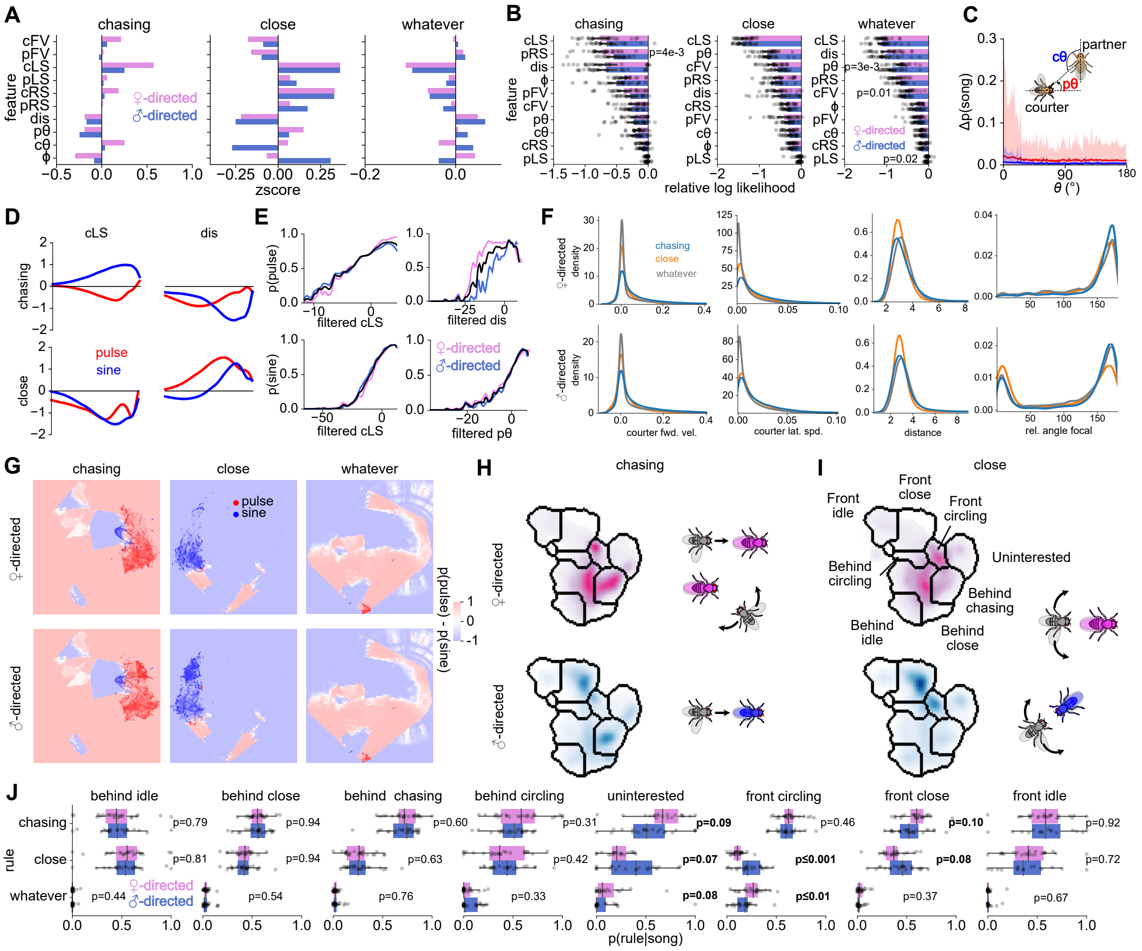

Figure S6: Modeling song patterning rules using HMM-GLM.

A The mean of z-scored features during the chasing, close, and whatever rules.

B Mean±std importance of behavioral features for each rule in the HMM-GLM, computed as the reduction (logarithmic scale) in model log-likelihood on the training data when the feature is randomly permuted (N=24 training trials each for female-directed and male-directed). Statistical significance values are only labeled where there is significant difference ($p<0.05$).

C Effect of courter position and courter orientation around the target on HMM-GLM predictions, computed as the difference in predicted probabilities of song by the model when changing the relative courter angle ($c\theta$) between 0 (head-directed) to 180deg (tail-directed) or relative partner angle ($p\Theta$) between 0 (focal oriented towards the target) to 180deg (focal oriented away from the target). The solid curves show the mean, and the shaded regions show the 95% CI. When changing $c\theta$, the mean change is virtually zero and the 95% CI is less than 0.04, indicating that the male's angular position around the target has a negligible impact on song patterns.

D Filters for courter lateral speed (cLS) and distance (dis) for pulse (red) and sine (blue) during the chasing and close rule.

E Probability of pulse (top) and sine (bottom) song as a function of filtered cLS and dis during the chasing and close rule. Curves are similar for female- (magenta) and male-directed (blue) singing, indicating that the mapping from behavioral features to song is independent of sex. Tuning curves for male and female directed pulse song are slightly offset for distance, due to differences in the distance distributions, with males tending to sing pulse song even at farther distances from females than from males (Fig. S4A–B).

F Probability density of courter forward velocity (cFV), courter lateral speed (cLS), distance (dis), and relative courter angle ($c\theta$) during the chasing, close, and whatever rules during female (top) and male-directed interactions (bottom). For both male and female targets, the courter male is fast and far during the chasing rule, close and slow during the close rule, and slow and far during the whatever rule. Additionally, towards male partners, the courter is predominantly placed near the head during close state.

G Filtered stimuli space visualized using UMAP embedding along with decision boundaries learned by the HMM-GLM visualized in the UMAP space (see Methods). The background colors show the difference between pulse and sine probabilities inferred by the model in the stimulus space.

H, I Social maps (left) and illustration of the interaction contexts (right) when the male uses the chasing (**H**) and close (**I**) rules during interactions with female (top row, magenta) and male (bottom row, blue) partners.

J Rule use for each social mode. The rule use depends on partner sex only in the modes associated with head interactions. Dots correspond to average value for each fly pair (N=30 pairs each of male-female and male-male pairs). Central mark indicates the median; the bottom and top edges of the box indicate the 25th and 75th percentiles, respectively. Whiskers extend to 1.5 times the interquartile range away from the box edges.

All p-values were computed using a two-sided Mann-Whitney U test.

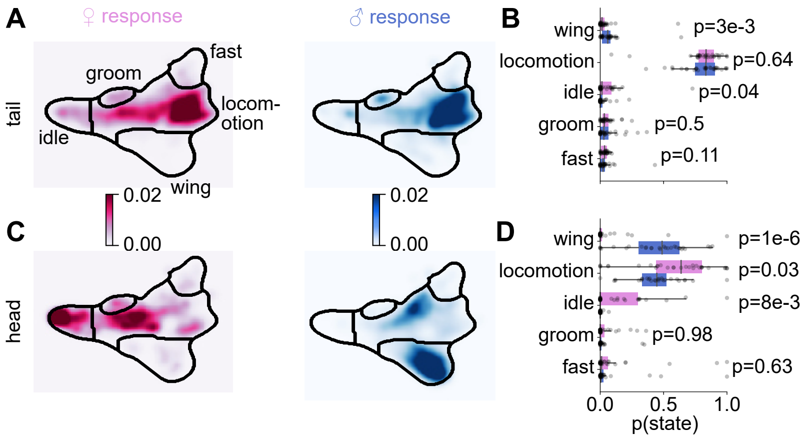

Figure S7: Partner's behavioral feedback drives differential song patterning
A–B Probability of different behaviors of female (right) and male (left) partner while the courter male is singing in the tail quadrant. The higher duration of sine song towards female partners during tail interactions (Fig. 2E) may be attributed to the increased idleness of females during such interactions which allow the male to interact more closely near female tail (Fig. 1H).
C–D Same as **A–B** but when the courter male is singing in the head quadrant. Differences in partner behavioral feedback are much more pronounced when the courter is in the head quadrant, driving differences in head-directed song patterning. Dots in **B, D** correspond to each fly pair (N=30 male-female and 30 male-male pairs). Central mark indicates the median; the bottom and top edges of the box indicate the 25th and 75th percentiles, respectively. Whiskers extend to 1.5 times the interquartile range away from the box edges. All p-values were obtained using a two-sided Mann-Whitney U test.

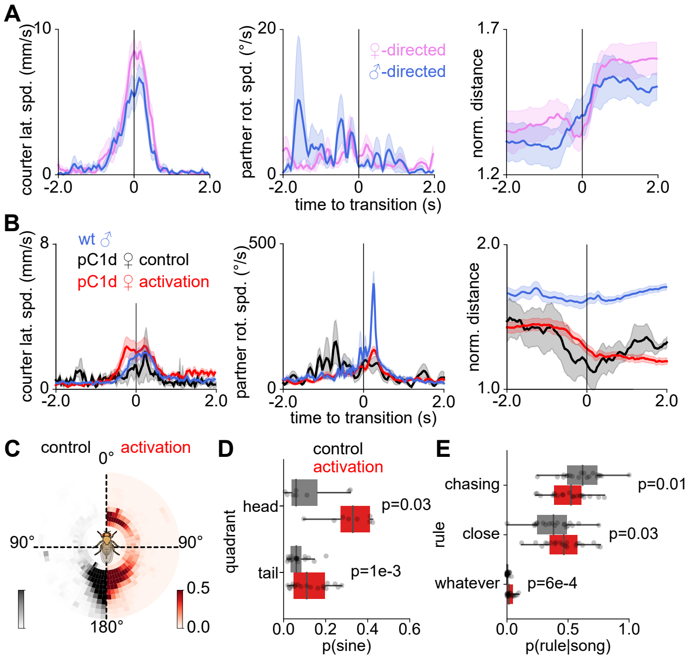

Figure S8: Manipulating partner's behavioral feedback modulates rule use and song patterning
A Courter lateral speed (left), partner rotational speed (middle), and normalized distance (right) during transitions into head interactions with an immobilized female or male partner. Both female- and male-directed transitions occur when the focal fly circles to the front of the target and thus leads to an increased distance between flies. Lines and shaded areas correspond to mean ±s.e.m. (14 transitions from 10 male-female pairs and 13 transitions from 7 male-male pairs). Distance is normalized by the body length of the immobilized partner.
B Courter lateral speed (left), partner rotational speed (middle), and distance to the partner (right) during transitions in head interactions with a female partner expressing csChrimson in aggression-inducing pC1d neurons. During activation (LED on), the partner turns back like a wild-type male partner reducing the distance between the flies. Lines and shaded areas correspond to mean ±s.e.m. (control: n=6 transitions from 5 pairs, and activation: n=57 transitions from 17 pairs).
C Position of a male courter around a pC1d-Chrimson female during control (grey, LED off) and activation (red, LED on).
D Fraction of sine song produced by male courter in the head and tail quadrants around a pC1d-Chrimson female during control (grey) and activation (red).
E Rule use when singing to a pC1d female partner during control (LED on) and activation (LED off). Dots in D and E correspond to each fly pair that interacted in the given quadrant during both control and activation (Head: N=8, Tail: N=19). For panels D–E, central mark indicates the median; the bottom and top edges of the box indicate the 25th and 75th percentiles, respectively. Whiskers extend to 1.5 times the interquartile range away from the box edges. p-values were obtained using Wilcoxon signed-rank tests.

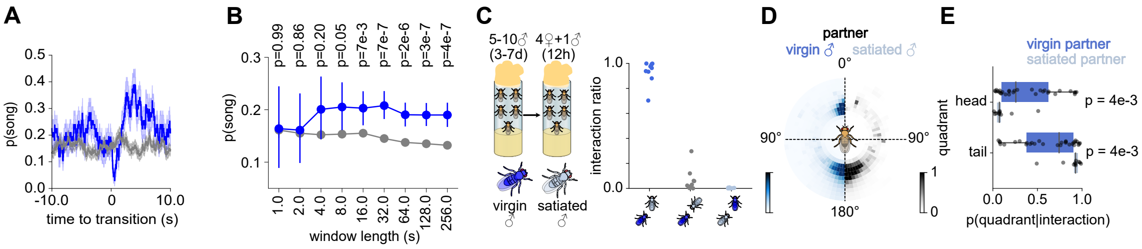

Figure S9: Slow song-driven buildup of the partner's social arousal drives head-interactions in males.
A Probability of courter singing around transitions to head interactions. The grey line shows song probability in randomly selected windows during tail interactions (head transitions: N=64 windows from 22 pairs; no transitions: N=2833 windows from 30 pairs).
B Amount of song (mean±std) in windows preceding head-interaction transitions (N=67, 64, 58, 58, 60, 61, 59, 57, 44 for 1 s, 2 s, 4 s, 8 s, 16 s, 32 s, 64 s, 128 s, and 256 s windows respectively). Grey line shows song in randomly selected windows without transitions (N=2134, 2035, 1914, 1821, 1737, 61, 59, 57, 44 for 1 s, 2 s, 4 s, 8 s, 16 s, 32 s, 64 s, 128 s, and 256 s windows respectively). Song is enriched only in longer windows, suggesting integration over extended timescales.
C Effect of sexual satiation on head interactions. Males were isolated after eclosion and group-housed for 3–7 days. Sexual satiation was induced by housing one male with four virgin females for ≥12 h before the experiment. Right: fraction of time spent in different configurations (N=10 pairs).
D Position of a male courting a naive (blue, left) or sexually satiated (black, right) male.
E Fraction of interaction time spent in head and tail quadrants when courting a mature virgin (blue) or satiated male (grey). Courters remain mostly near the tail of satiated partners.
p-values in B, E were computed using two-sided Mann–Whitney U tests; Lines and shaded areas show mean ± s.e.m.; dots in E represent fly pairs (virgin: N=30 pairs; satiated: N=10 pairs).
