# Supplementary material for: Sex-specific behavioral feedback modulates sensorimotor processing and drives flexible social behavior
Source: Nat Commun. 2026 May 4;17:4026. doi: 10.1038/s41467-026-72057-9 (PMC13139495; doi:10.1038/s41467-026-72057-9)
Supplement: Supplementary file 4 — Description of Additional Supplementary Files [file 41467_2026_72057_MOESM4_ESM.pdf]

Description of Additional Supplementary Files

File Name: Supplementary video 1

Description: Example videos of male-female (left 3x3 panels) and male-male (right 3x3 panels) interacting in the "behind chasing" social mode. The male focal fly is behind a female or male partner and pursuing the partner. Each video is looped 10 times. The videos are centered at the midpoint of the thoraces of the two flies and cropped such that each boundary is at least 100px from the nearest fly's thorax. When the cropping boundary is outside the original video, rest of pixels are filled in black. Video playback at normal speed (MP4, 8486 KB).

File Name: Supplementary video 2

Description: Example videos of male-female (left 3x3 panels) and male-male (right 3x3 panels) interacting in the "behind circling" social mode. The male focal fly is placed behind the male or female target and is moving towards the target abdomen. Each video is looped 10 times. The videos are centered at the midpoint of the thoraces of the two flies and cropped such that each boundary is at least 100px from the nearest fly's thorax. When the cropping boundary is outside the original video, rest of pixels are filled in black. Video playback at normal speed (MP4, 4915 KB).

File Name: Supplementary video 3

Description: Example videos of male-female (left 3x3 panels) and male-male (right 3x3 panels) interacting in the "behind close" social mode. The male focal fly is placed close behind the male or female target and the both flies are slow. Each video is looped 10 times. The videos are centered at the midpoint of the thoraces of the two flies and cropped such that each boundary is at least 100px from the nearest fly's thorax. When the cropping boundary is outside the original video, rest of pixels are filled in black. Video playback at normal speed (MP4, 3486 KB).

File Name: Supplementary video 4

Description: Example videos of male-female (left 3x3 panels) and male-male (right 3x3 panels) interacting in the "behind idle" social mode. The male focal fly is placed behind the male or female target and the both flies are idle. Each video is looped 10 times. The videos are centered at the midpoint of the thoraces of the two flies and cropped such that each boundary is at least 100px from the nearest fly's thorax. When the cropping boundary is outside the original video, rest of pixels are filled in black. Video playback at normal speed (MP4, 2138 KB).

File Name: Supplementary video 5

Description: Example videos of male-female (left 3x3 panels) and male-male (right 3x3 panels) interacting in the "front circling" social mode. The male focal fly is placed near the front of a male or female target. During male-female interactions, the female is stationary and male circles in her front. During male-male interactions, both males circle each other. Each video is looped 10 times. The videos are centered at the midpoint of the thoraces of the two flies and cropped such that each boundary is at least 100px from the nearest fly's thorax. When the cropping boundary is outside the original video, rest of pixels are filled in black. Video playback at normal speed (MP4, 6901 KB).

File Name: Supplementary video 6

Description: Example videos of male-female (left 3x3 panels) and male-male (right 3x3 panels) interacting in the "front close" social mode. The male focal fly is placed near the front of and close to a male or female target, and the movement is slow. During malemale interactions, the males face each other head-to-head. During male-female interactions, the males are placed with an offset from female head. Each video is looped 10 times. The videos are centered at the midpoint of the thoraces of the two flies and cropped such that each boundary is at least 100px from the nearest fly's thorax. When the cropping boundary is outside the original video, rest of pixels are filled in black. Video playback at normal speed (MP4, 3076 KB).

File Name: Supplementary video 7

Description: Example videos of male-female (left 3x3 panels) and male-male (right 3x3 panels) interacting in the "front idle" social mode. The male focal fly is placed near the front of a male or female target, and in most cases, both flies are idle. Each video is looped 10 times. The videos are centered at the midpoint of the thoraces of the two flies and cropped such that each boundary is at least 100px from the nearest fly's thorax. When the cropping boundary is outside the original video, rest of pixels are filled in black. Video playback at normal speed (MP4, 1929 KB).

File Name: Supplementary video 8

Description: Example videos of male-female (left 3x3 panels) and male-male (right 3x3 panels) interacting in the "uninterested" social mode. The flies are away from each other or facing in opposite directions. The videos are centered at the midpoint of the thoraces of the two flies and cropped such that each boundary is at least 100px from the nearest fly's thorax. When the cropping boundary is outside the original video, rest of pixels are filled in black. Video playback at normal speed (MP4, 5726 KB).

File Name: Supplementary video 9

Description: Example videos of transitions from tail-directed interactions to head-directed interactions in male-female pairs. The head-directed interactions occur when the female target is stationary and focal male circles to her front maintaining a distance. The videos are centered at the midpoint of the thoraces of the two flies and cropped 150px from the center to each side. When the cropping boundary is outside the original video, rest of pixels are filled in black. Video playback at normal speed (MP4, 1495 KB).

File Name: Supplementary video 10

Description: Example videos of transitions from tail-directed interactions to head-directed interactions in male-male pairs. The head-directed interactions occur when the male target is stationary and male target turns back to the focal male reducing the speed of focal male and distance between the flies. The videos are centered at the midpoint of the thoraces of the two flies and cropped 150px from the center to each side. When the cropping boundary is outside the original video, rest of pixels are filled in black. Video playback at normal speed (MP4, 2503 KB).
